# Scrolling for science: Assessing the quality and accuracy of Crohn’s disease-related content on Instagram reels

**DOI:** 10.1371/journal.pone.0350430

**Published:** 2026-06-17

**Authors:** Sankirth Madabhushi, Ami Patel, Samantha Tse-Kang, Andrew Liu, Dhruval Amin, Abbas Rupawala

**Affiliations:** 1 Department of Medicine, UMass Chan Medical School, Worcester, Massachusetts, United States of America; 2 UMass Chan Medical School, Worcester, Massachusetts, United States of America; 3 Department of Medicine, Division of Gastroenterology, UMass Chan Medical School, Worcester, Massachusetts, United States of America; University of Basel Institute for Biomedical Ethics: Universitat Basel Institut fur Bio- und Medizinethik, SWITZERLAND

## Abstract

**Background:**

There is a concerning trend of misinformation of healthcare related content on social media. Recent studies have examined themes and narratives about Crohn’s disease but have not quantitatively assessed the accuracy and quality of content on Instagram Reels. Our aim was to assess the quality and accuracy of Instagram Reels about Crohn’s disease and examine differences in content by type of creator, from medical professionals to lay individuals.

**Methods:**

Seventy-eight top-viewed English-language Instagram Reels tagged with “#crohns” were evaluated. Videos were categorized by creator and content type. Two reviewers evaluated each video for accuracy and quality using an adapted harm/benefit score and the Journal of the American Medical Association (JAMA) benchmark criteria, respectively.

**Results:**

Seventeen percent of videos were created by medical professionals and 83% by non-medical users. Educational content was significantly more common among medical professionals than other content creators (62% vs 23%; *P* = 0.005). No significant correlation was found between engagement metrics and either JAMA or harm/benefit scores. Medical professionals had significantly higher JAMA scores than non-medical users (2.5 vs 2, *P* < 0.001), but there was no significant difference in harm/benefit scores between groups (0 vs 0, *P* = 0.9601). Videos offering medical advice had the lowest median harm/benefit score (−1), with frequent misinformation noted. Forty-two percent of harmful videos were created by medical professionals.

**Conclusions:**

The average Instagram Reel about Crohn’s disease was of moderate quality and neutral impact. Accuracy or quality was unrelated to video popularity. While videos by medical professionals had higher JAMA scores, this did not correspond to greater accuracy. Medical advice videos by medical professionals were not more accurate than those by non-medical creators, and multiple harmful videos were created by medical professionals, underscoring the need for critical evaluation of Crohn’s disease-related social media content.

## Introduction

Crohn’s disease (CD) is an inflammatory bowel disease (IBD) affecting millions worldwide, with a prevalence of 0.3% in North America [1]. CD is a progressive disease involving both the small bowel and colon, with several long-term complications including strictures, fistulae, and abscesses, which can lead to significant morbidity.

The digital landscape has transformed how individuals access health information. Instagram is a visually driven platform focusing on photos and short form videos with over three billion users [2]. It is extensively utilized for health discussions across a variety of groups, from medical professionals, to lay people, to organizations like the Centers for Disease Control and Prevention and the National Institutes of Health. However, the accuracy of medical information on social media is highly variable, not peer-reviewed, and sometimes misleading.

Medical content on social media is widespread, and studies suggest that a significant proportion of internet users use platforms like Instagram, TikTok, Facebook, and YouTube as sources of health information [3–6]. For instance, research on allergic contact dermatitis found that while patient-generated content was abundant, its medical accuracy was often low [7]. Similarly, a study on Ebola imagery revealed that 78% of images were irrelevant or misleading [8]. Furthermore, Instagram has been implicated in promoting harmful behaviors, including tobacco and alcohol use, and contributing to eating disorders [9–13]. The pervasive spread of health misinformation has emerged as a critical public health challenge, formally recognized by the U.S. Surgeon General’s 2021 advisory that characterized it as an “infodemic” [14]. Defined as information that is false, inaccurate, or misleading based on the best available evidence, infodemics erode public trust in authoritative health sources and directly harm individual health outcomes [15]. The rapid dissemination of such content, particularly across digital platforms, is exacerbated by algorithms that prioritize engagement over accuracy [16].

The specific impact of Instagram in the context of CD remains under explored. Existing research has examined IBD treatments discussed on TikTok, themes within adolescent IBD support communities, and the platform’s potential for patient education [6,17,18]. However, the accuracy and quality of CD-related social media information are poorly characterized, highlighting an urgent need for further research. Here, we evaluate the quality and accuracy of Crohn’s disease tagged content on Instagram Reels and examine differences in content by type of creator.

## Materials and methods

### Search strategy and eligibility criteria

**Fig 1:**
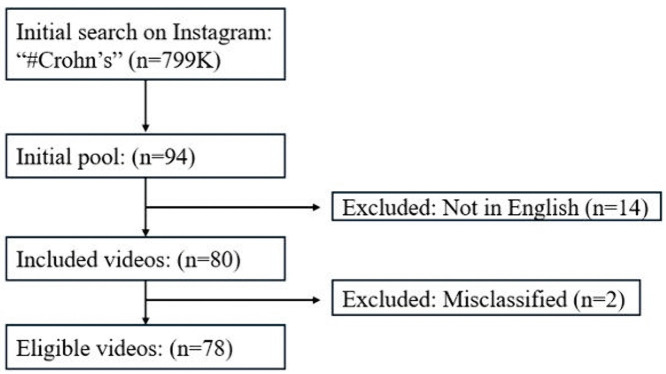
Video selection flowchart. Flowchart illustrating the inclusion and exclusion criteria for videos to be analyzed.

A new Instagram account was created to reduce bias from any prior searches that could influence social media algorithms. A search on Instagram Reels with the hashtag “#crohns” yielded 799,000 results that were a combination of posts and reels. The top 94 videos were initially identified and selected on January 21, 2025, over 2 hours to minimize reshuffling of “top” Reels and limit bias due to personalization and targeted videos from Instagram’s dynamic ranking and personalization algorithms. To avoid selection bias, we included all videos presented by Instagram under “Top Reels” at the time of our unbiased capture, regardless of view count. Inclusion criteria for the study included the following: 1) English language, and 2) contains information in the form of audio and/or text. Of the initial videos, 14 were excluded because they were not in English, and two were excluded because they lacked sufficient audio or text. The remaining 78 videos met inclusion criteria and were eligible for further analysis (Fig 1).

### Data management

Study data were collected and managed using REDCap (Research Electronic Data Capture) hosted at UMass Chan Medical School. REDCap is a secure, web-based software platform designed to support data capture for research studies, providing 1) an intuitive interface for validated data capture; 2) audit trails for tracking data manipulation and export procedures; 3) automated export procedures for seamless data downloads to common statistical packages; and 4) procedures for data integration and interoperability with external sources [19,20]. Data management strategies were adapted from a previous publication studying social media health trends [21].

For each video that met the inclusion criteria, data were recorded on the date of collection, which included the URL, date posted, number of views, likes, comments, and shares. Uploader type was defined as the following: medical professional (individuals holding an MD/DO/MBBS, PhD, MPH/MS, RN, NP/PA, RD, or PharmD degree); professional organization; lay individual (non-medical professional with <10,000 followers); and influencer (non-medical professional with >10,000 followers) [21]. Each video was categorized by type, which included educational, marketing, product review, video log (vlog), interview, or motivational. If a video fell into multiple categories, the best fit was identified. Content intention was also noted as either medical advice, lifestyle, comedy, marketing, advocacy, patient experience, or charity. Video content, particularly if educational, was validated through either a literature review or consultation with physicians practicing gastroenterology and/or an IBD specialist. Two independent reviewers assessed the videos’ quality and accuracy using two independent scoring systems, and the mean scores were used. Videos with large discrepancies in scores between the original two reviewers were assessed with a third reviewer. The de‑identified metadata are publicly available as Supplementary S1 Table and include Record ID, number of views, number of comments, number of likes, number of shares, video type, content intention, uploader type, mean harm/benefit score, and mean JAMA score. No Instagram media files were downloaded. Video links may be available upon reasonable request.

### Tools to assess harm, quality and validity of videos

Using a literature-based harm/benefit scale adapted to our context, we assigned each video −1, 0, or 1 [21]. A score of −1 denoted a video that contained misleading, incorrect, or nonfactual information that could promote dangerous advice related to CD and potentially harm the viewer. A score of 0 indicated a video that was neither harmful nor beneficial (e.g., a comedy or lifestyle video that is not sharing nonfactual information). A score of 1 denoted a video that provided accurate and helpful information related to CD that could improve the viewer’s understanding of the topic or promote healthy behaviors.

The Journal of the American Medical Association (JAMA) criteria score was used to assess the quality and validity of a video [22]. This score is based on four JAMA criteria: authorship, attribution, disclosure and currency. Each component had a score of either 0 or 1, with a combined maximum of 4 points. A score of 1 for authorship indicated the presence of listed authors and/or contributors with clear indication of their position/profession in the video and/or profile. A score of 1 for attribution demonstrated clear listing of references, sources, and/or copyright information. A score of 1 for disclosure was given to videos with clear disclosure of video ownership, sponsorship, advertising and potential conflicts of interest. A score of 1 for currency indicated clear posting of video upload date.

### Statistical analysis

Descriptive statistics were performed using univariate analysis with chi-squared, two-tailed Mann–Whitney U tests, and Kruskal-Wallis H test with post-hoc Dunn’s tests and Bonferroni correction performed when applicable. All categories of videos not created by a medical professional were pooled as “non-medical professional.” Continuous variables were presented as medians with interquartile ranges [first quartile (Q1)–third quartile (Q3)]. To determine the correlation between daily views vs. JAMA score, daily views vs. harm/benefit score, and JAMA vs harm/benefit scores, random intercept linear regression modeling was performed. To reduce the influence of the video’s age on raw view totals, we normalized number of views by computing views per day = total views ÷ (days since posting + 1). The “+1” prevents division by zero for videos posted the same day as our capture. Spearman’s rank correlation test was then used to determine correlation among variables. The Spearman’s rank correlation coefficient (r) for each comparison is denoted within the corresponding figures. The significance level was set at *P* < 0.05. Statistical analyses were performed using GraphPad Prism (v10.0.3), Stata (v16.1), and online statistical calculators (Social Science Statistics; https://www.socscistatistics.com).

### Technology and ethics statements

This project was reviewed by the UMass Chan Medical School Institutional Review Board (IRB), who determined that this is not research involving human subjects. Therefore, IRB review, approval, and informed consent was not required. We recorded only content that was publicly accessible at the time of capture. We used OpenAI’s ChatGPT to assist with revising text. No data collection, generative editorial work and autonomous content creation, coding, analysis, or interpretation was performed by the tool. All AI‑generated text was reviewed and approved by the authors. No automated scraping was performed, and all videos were accessed and reviewed manually by the authors. We did not access private data like private profiles, messages, or otherwise restricted content. We do not redistribute copyrighted media files. Original videos are third‑party content hosted on Instagram and subject to Instagram’s terms. We only utilized uploader‑reported information (as publicly displayed). To the best of our knowledge, these procedures were consistent with Instagram’s publicly available terms.

## Results

### Baseline video characteristics

**Fig 2:**
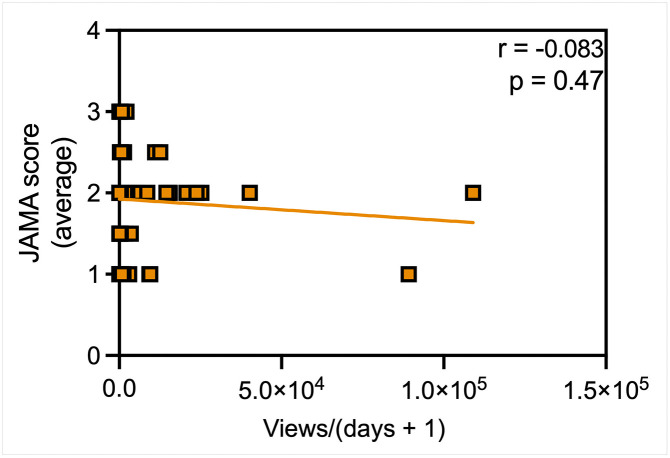
Views vs JAMA score. Scatter plot showing no correlation between daily views and JAMA scores.

**Fig 3:**
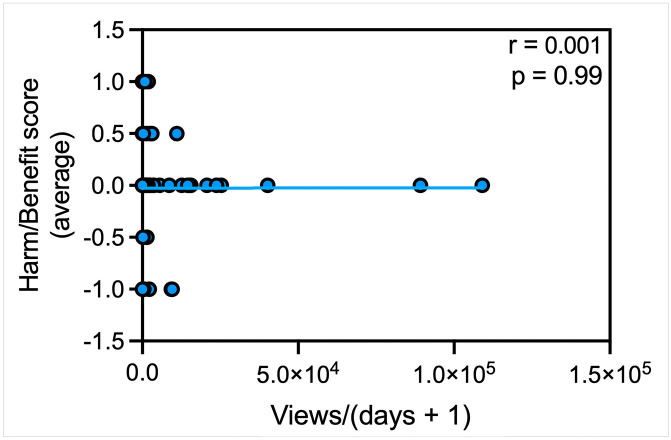
Views vs harm/benefit score. Scatter plot showing no correlation between daily views and harm/benefit scores.

Of the 78 videos analyzed, the majority (n = 65, 83%) were created by non-medical professionals, while 13 (17%) were by medical professionals (Table 1). The proportion of educational videos differed by creator type, with a higher proportion created by medical professionals compared with non–medical professionals (62% vs 23%; χ^2^ = 7.71; *P* = 0.005). Non-medical creators were the most frequent creators overall with their content predominantly focusing on lifestyle (51%) and patient experience (34%). Regarding video type across all videos, vlogs constituted the largest category (47%), followed by educational (30%) and marketing (14%) videos. Across all videos, content intended to deliver lifestyle information was the most prevalent (46%), with patient experience (28%) and medical advice (22%) also being common (Table 1). In terms of engagement, videos by non-medical creators had fewer average daily views than those by medical creators (492 [85–2,441] vs 738 [258–4160], U = 386, *P* = 0.625), though these differences were not significant. Furthermore, no significant correlation was found between a video’s average daily view count and its JAMA score (r = −0.083, *P* = 0.471) (Fig 2) or harm/benefit score (r = 0.001, *P* = 0.995) (Fig 3). To account for the potential effect of low-view video outliers, we performed additional sensitivity analyses that also showed no significant correlation (S1–S4 Figs).

**Table 1:** Engagement and quality/accuracy measures by creator type.

	Overall n = 78	Medical Professionals n = 13	Non-medical influencers n = 20	Lay People n = 41	Professional Organizations n = 4
Views (cumulative) — median	5330	6709	64100	1703	202
Shares (cumulative) — median	5.50	9	18	1	0.5
Harm Score — median	0	0	0	0	0
JAMA score— median	2	2	2	2	1
**Video Type — no. (%)**					
Educational	23 (29.5)	8 (61.5)	4 (20.0)	10 (24.4)	1 (25.0)
Interview	1 (1.3)	0	0	1 (2.4)	0
Marketing	11 (14.1)	5 (38.5)	2 (10)	3 (7.3)	1 (25.0)
Motivational	6 (7.7)	0	1 (5)	3 (7.3)	2 (50.0)
Vlog	37 (47.4)	0	13 (65)	24 (58.5)	0
**Type of content— no. (%)**					
Medical Advice	17 (21.8)	7 (53.8)	4 (20.0)	5 (12.2)	1 (25.0)
Lifestyle	36 (46.2)	1 (7.7)	12 (60.0)	21 (51.2)	2 (50.0)
Comedy	8 (10.3)	2 (15.4)	2 (10.0)	4 (9.8)	0
Marketing	16 (20.5)	7 (53.8)	3 (15.0)	5 (12.2)	1 (25.0)
Advocacy	3 (3.8)	2 (15.4)	0	1 (2.4)	0
Patient experience	22 (28.2)	0	8 (40)	14 (34.1)	0
Charity	3 (3.8)	3 (7.3)	0	0	0

Engagement metrics and quality/accuracy measures, with video and content categories, by creator type.

### JAMA scores

The median JAMA score across all videos was 2 [1.5–2]. When examining content intention types, advocacy and marketing videos had the highest median JAMA scores (2.5 and 2.25, respectively) (Table 2). Notably, videos created by medical professionals had significantly higher JAMA scores compared to those made by non-medical professionals (2.5 [2–2.5] vs 2 [1.5–2], U = 418.5, *P* < 0.001). However, the Kruskal-Wallis H test showed no significant difference in JAMA scores across different video types (H = 8.11; *P =* 0.088) or different content intentions (H = 10.58; *P* = 0.102). Additionally, the median JAMA score was the same (2) for videos with negative harm/benefit ratings and those with neutral or positive scores (U = 395.5, *P* = 0.999). Finally, within the medical advice content intention category, there was no significant difference in JAMA scores between medical professionals and other creators (2.5 [2.25–2.75] vs 2 [1.125–2], U = 18.5, *P* = 0.119).

**Table 2:** Harm/benefit and JAMA scores by content type.

Type of Video Content	Median Harm/Benefit Score	Median JAMA Score
Medical Advice	−1 [−1, 0]	2 [1.5, 2.5]
Lifestyle	0 [0, 0]	2 [1.5, 2]
Comedy	0 [0, 0.125]	2 [2 2]
Marketing	0 [−1, 0]	2.25 [2, 2.625]
Advocacy	0.5 [0.25,0.75]	2.5 [1.75, 2.75]
Patient Experience	0 [0,0]	2 [1.625, 2]
Charity	0 [0,0]	2 [1.75-2]

Average harm/benefit score and JAMA score according to type of video content.

### Harm/benefit scores

**Fig 4:**
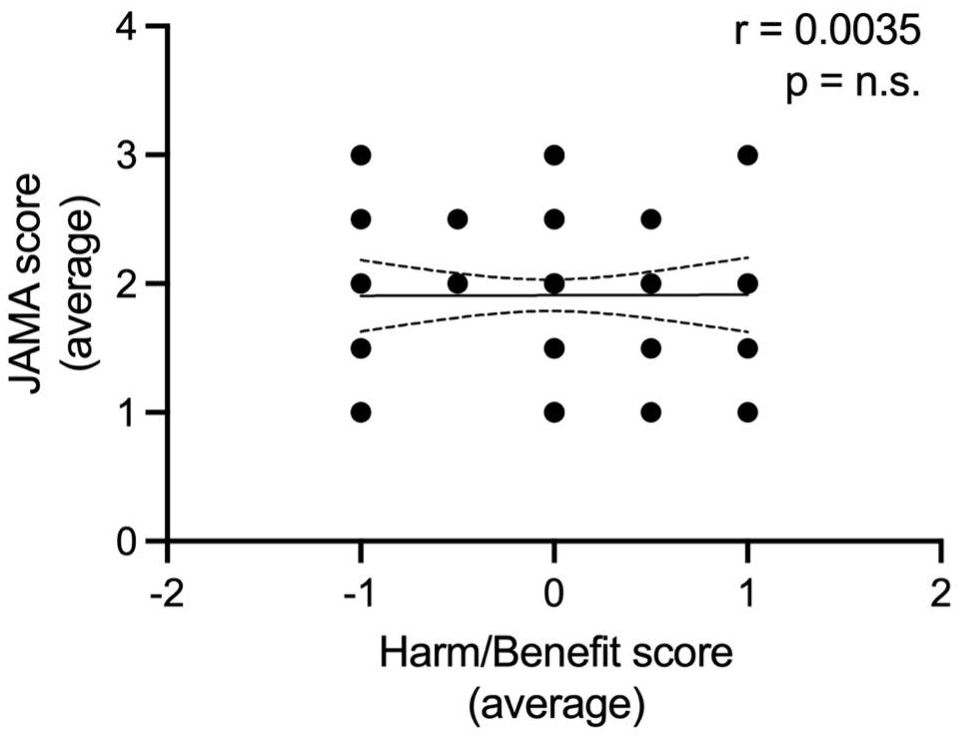
Harm/benefit vs JAMA score. Scatter plot showing no correlation between harm/benefit scores and JAMA scores. n.s. = not significant.

The median harm/benefit score across all videos was 0 [0,0]. We found no significant difference in harm/benefit scores between videos created by medical professionals and those by non-medical creators (0 [−0.50, 0.50] vs 0 [0, 0], U = 418.5, *P* = 0.960). Additionally, harm/benefit scores did not correlate with JAMA scores (r = 0.004, *P* = 0.976) (Fig 4). Approximately one-third of all videos had a positive harm/benefit score (> 0). When examining content intention types, advocacy videos had the highest median harm/benefit scores (0.50) (Table 2). Conversely, medical advice videos had the lowest median harm/benefit score (−1) (Table 2). Among videos with a negative harm/benefit score, 42% (5/12) were created by medical professionals.

We found no significant difference in overall harm/benefit scores across the different video types (H = 7.44; *P* = 0.114). However, a significant difference was observed in harm/benefit scores across video content intentions (H = 25.6; *P* < 0.001). Post-hoc Dunn’s tests, with Bonferroni correction, revealed that medical advice videos exhibited significantly different harm/benefit scores when compared to lifestyle videos (−1 [−1, 0] vs. 0 [0, 0], *P* < 0.001), comedy videos (−1 [−1, 0] vs. 0 [0, 0.125], *P* = 0.002), advocacy videos (−1 [−1,0] vs 0.5 [0.25,0.75], *P* = 0.001), and patient experience videos (−1 [−1, 0] vs. 0 [0, 0], *P* < 0.001). No other pairwise comparisons reached statistical significance. Within the medical advice category, harm/benefit scores did not differ significantly between videos created by medical professionals and those created by others (−0.5 [−1, 0.25] vs −1 [−1, − 0.25], U = 24, *P* = 0.308). Specific content is further detailed in Table 3.

**Table 3:** Categorized list detailing videos with a harm/benefit score of < 0.

Primary Category	Summary of Video Content	Rationale	Creator Type	Reference:
Clinical Data Misinterpretation	Creator interprets an abdominal X-ray as showing signs of inflammation with concomitant blood work to suggest a parasitic etiology and advertises services	Diagnosing GI pathologies is not within a chiropractor’s scope of practice. Abdominal X-rays lack the specificity required to identify inflammation or validate parasitic etiologies.	Lay individuals	[23,24]
	Misappropriation of endoscopic media showing IBD, parasitic infections, and poor prep to promote gut-balancing products	Gut balancing microbiome products are not guideline-supported treatments for IBD and parasitic infections.	Non-medical influencers	[25,26]
	Creator performs a GI work up for a patient with a previous diagnosis of IBD and instead suggests allergies, infections, and nonspecific inflammation as the true causes.	Diagnosing and treating IBD is not within a chiropractor’s scope of practice. Biomarkers alone are insufficient for diagnosing IBD, allergies, or infections.	Non-medical influencers	[27]
Incorrect pathophysiology	Claims IBS causes Hashimoto’s thyroiditis and bloating is caused by microbiome imbalance and a lack of “good” bacteria	Interchanges IBD with IBS, and there is no conclusive evidence that IBS and bloating causes Hashimoto’s thyroiditis	Non-medical influencers	[28]
	Claims lack of microbiome diversity causes IBD and Hashimoto’s thyroiditis	There is inconclusive evidence whether dysbiosis causes IBD or is a result of IBD. There is no consensual evidence that a lack of microbiome diversity causes Hashimoto thyroiditis.	Non-medical influencers	[29]
Non-Standard Diagnostic Strategies	Claims use of advanced tests (Trio SIBO breath, GI-MAP stool, leaky gut panels, SIBO aspiration) can diagnose the cause of IBS symptoms.	Partially true, although not associated with Crohn’s disease. The relationship between SIBO and symptoms associated in those without obvious predisposing factors is weak. Comprehensive stool and microbiome tests lack clinical validation for routine use. Leaky gut panels are primarily research rather than clinically validated tools.	Medical professionals	[30]
	Suggests use of microbiome mapping to diagnose IBD	Use of microbiome mapping to diagnose IBD is not guideline-supported. There are studies uncovering universal IBD-associated bacteria; however, no noninvasive tool has achieved regulatory approval for diagnostic purposes.	Medical professionals	[25,31–33]
	States fructose intolerance is more prevalent than lactose intolerance without further explanation and recommends advanced testing to diagnose IBS.	Partially true, although not associated with Crohn’s disease. Fructose intolerance is more prevalent than lactose intolerance among patients with disorders of gut-brain interaction, but this may not be reflective of the general population. Current guidelines suggest a stepwise approach starting with empirical dietary restriction rather than immediate testing as suggested.	Medical professionals	[34–37]
	Misrepresents CD etiology and recommends nonstandard functional blood chemistry diagnostics	There is no conclusive evidence supporting non-gastroenterologists using functional blood chemistries to diagnose IBD.	Lay individuals	[26]
Non-Standard Therapeutic Strategies	Promotes BPC-157, KPV, and larazotide as effective treatments for IBD, IBS, Celiac’s disease	These substances are not FDA-approved for human consumption	Medical professionals	[38]
	Claims using integrative colonoscopy preps with microbiome restoration ingredients can preserve gut health	Partially true. Preliminary research suggests that adding green tea or probiotics peri-colonoscopy may reduce side effects of bowel prep. This is not endorsed by major gastroenterology societies, and osmotic preps (PEG, sodium sulfate, etc.) remain the standard.	Medical professionals	[39–41]
	Mischaracterizes IBD and IBS and advises management with only vital sign monitoring and unproven functional therapies	IBD and IBS are two distinct conditions, and IBD should not be treated with functional therapies and monitoring vital signs.	Professional organization	[26]

List of all twelve videos with a harm/benefit score of < 0 organized by type of misinformation. Abbreviation key: GI, gastrointestinal; IBD, inflammatory bowel disease; IBS, irritable bowel syndrome; SIBO, small intestinal bacterial overgrowth; GI MAP, Gastrointestinal Microbial Assay Plus; CRP, C-reactive protein; PEG, polyethylene glycol; FDA, United States Food and Drug Administration; BPC‑157, Body Protection Compound‑157; KPV, lysine–proline–valine peptide.

## Discussion

To our knowledge, this study is the first to evaluate the quality and accuracy of Crohn’s disease-related content on Instagram Reels. Overall, the average video on Instagram Reels related to CD in our study was of moderate quality and neutral in impact. Videos about CD are a prevalent topic on social media, with some videos having up to 6 million views. Most videos were created by non-medical professionals, and only one-third of the collected videos provided beneficial information for viewers. The other videos delivered neutral or even harmful information for viewers. Notably, videos created by medical professionals were no more popular than those created by non-professionals. Similarly, videos that improve understanding of CD or high-quality videos are no more likely to gain popularity compared to low-quality or harmful ones. Lay individuals predominantly created videos about lifestyle and CD.

Notable disparities were seen when content was stratified by intention. Lifestyle and patient experience videos were mostly neutral, meaning that they were neither overtly harmful nor strongly health-promoting. This may be because most lifestyle content creators are not healthcare professionals. While advocacy and comedy videos were most likely to spread beneficial information about CD, videos intended to deliver medical advice were among the most likely to spread misinformation. This suggests that a substantial amount of CD content regarding medical advice, regardless of the creator’s qualifications, may be questionable. Thus, providers should be aware of the influence social media has on IBD patients and support them in making informed decisions. Studies investigating the public health implications of misinformation on social media are still being conducted, and they will be key in curbing misinformation [21].

The misinformation seen in CD related reels was not only frequent, but also clinically significant. For example, some videos misrepresented IBD, irritable bowel syndrome, small intestinal bacterial overgrowth, and food intolerances as interchangeable conditions with CD and other autoimmune conditions (Table 3). Some creators were quick to attribute nonspecific gastrointestinal symptoms, such as bloating or diarrhea, to vague “gut microbiome imbalances,” suggested therapies not approved by the U.S. Food and Drug Administration for human use, or advertised their own functional medicine strategies over established therapies and diagnostics. Overall, social media may contain a breadth of misinformation, from irrelevant or tangential information to explicitly harmful recommendations. Future longitudinal studies and detailed qualitative analyses of CD related content may better assess the real-world impact of social media health content on patient knowledge and behavior [42,43].

Interestingly, while medical professionals were significantly more likely to create educational-type videos and scored higher on JAMA quality metrics, their content was not associated with greater factual accuracy or benefit compared to lay individuals. In fact, 42% of all harmful or misleading videos were created by medical professionals. Given the limited number of videos by medical professionals in our sample and that we were only able to observe those active on social media, this should not be interpreted as representative of medical professionals as a whole. Rather, society might benefit from specific studies focusing on content created by medical professionals. Our findings underscore the need for clearer guidelines for health communication on social media and updated tools to assess online health information in the age of short-form video platforms.

This exploratory study is subject to several limitations. The JAMA criteria were published in 1997 to evaluate the quality of health information on websites, but it may not fully capture the quality and accuracy relevant to short-form video content on platforms like Instagram. In our study, no videos met all four criteria, and 9% met three of four criteria [22,44,45]. Thus, it is not an ideal tool to comprehensively assess both accuracy and quality on social media, yet it is one of the most frequently used criteria to evaluate digital content due to a paucity of other validated frameworks. Additionally, an adapted harm/benefit scale applied here, though informative, is not standardized for social media health content and may be subject to interpretation. The inherent diversity within the #crohns videos that span from patient experience and lifestyle content to medical advice on specific clinical manifestations of CD can influence overall harm/benefit scores. This may affect nonclinical content scoring higher than medical‑advice content, which is innately more prone to misinformation. Thus, it is crucial to consider the development of updated frameworks to assess the quality and accuracy of online health content tailored to today’s expansive digital and social landscape. To expand upon this preliminary exploration of the informational landscape of health-related content on social media, perhaps future research could leverage Natural Language Processing and machine learning to scale the manual coding framework established in this study and analyze a greater volume of content. There may be potential for social media platforms to improve credibility and quality measures for health content, potentially with the assistance of artificial intelligence, to guide users toward trustworthy content. In the future, this may inform platform algorithms, as suggested by recent literature on the need for enhanced regulation and quality control in digital health communication [6,46,47].

## Supporting information

S1 Table Corresponding data for videos analyzed. (XLSX)

S1 Fig Views/(days + 1) vs JAMA score excluding videos with <1000 views. Scatter plot showing no significant correlation between number of Views/(days + 1) and JAMA scores.
(TIF)

S2 Fig Views/(days + 1) vs JAMA score excluding videos with <10000 views. Scatter plot showing no significant correlation between number of Views/(days + 1) and JAMA scores.
(TIF)

S3 Fig Views/(days + 1) vs Harm/Benefit score excluding videos with <1000 views. Scatter plot showing no significant correlation between number of Views/(days + 1) and Harm/Benefit scores.
(TIF)

S4 Fig Views/(days + 1) vs Harm/Benefit score excluding videos with <10000 views. Scatter plot showing no significant correlation between number of Views/(days + 1) and Harm/Benefit scores.
(TIF)
